# 

**Published:** 1805-10-01

**Authors:** 


### To CORRESPONDENTS.

Communications are received from Dr. Arneman, Dr. Cuming, Dr. Forbes, Dr. Gillespie, Dr. Hendy, Mr. Stevenson, Mr. T. Walworth, and Juvenis.

